# Red blood transfusion in patients undergoing cardiac surgery

**DOI:** 10.1007/s12471-014-0627-8

**Published:** 2014-11-27

**Authors:** L. Noyez

**Affiliations:** Department of Cardio-Thoracic Surgery, Heart Center, Radboud University Nijmegen Medical Center, PO Box 9101, 6500 HB Nijmegen, the Netherlands

The use of allogeneic red blood cells (RBC) is commonplace in cardiac surgery, with reported transfusion rates ranging from 5 to 90 %. [1–4] Despite advantages, RBC transfusion is associated with well-described adverse outcomes. [1–4] Transfusion of RBCs is not only associated with an increased perioperative mortality and morbidity, however; it also results in a longer ICU stay, total hospital stay and increased costs. [2] Even the long-term results are influenced by perioperative RBC transfusions. Koch et al. found a significantly reduced 6-month and late survival in patients undergoing an isolated CABG with transfusion of RBCs. [1] Not only the question of RBC transfusion, but also the number of units of transfused RBCs and the method and duration of storage of RBCs is important. [3, 4]

It is no surprise that several studies focus on blood conservation methods and a more judicious use of RBCs, with as prerequisite the identification of preoperative variables associated with an increased need for transfusion. [3, 4] Female gender, impaired left ventricular function, low preoperative haemoglobin, risk and complexity of the operation are the most identified variables with an increased risk for RBC transfusion [3, 4] However, even with the routine use of current blood conservation methods, a proportion of patients undergoing cardiac surgery continue to receive RBC transfusion and are hence exposed to its associated risk [1–4]. There is, therefore, an ongoing need to study further blood conservation methods.

In their recent study, [5] Haanschoten et al. focus on reducing the immediate availability of RBCs in cardiac surgery. Since March 2010, the No Elective Red Cells (NERC) program is being used. The only change for their current study is that RBCs are now not directly available in the operating theatre and, when needed, the local blood bank can deliver the cross-matched RBCs within 20 min. So, the aim of this new strategy is to reduce the number of units of RBCs returned to the laboratory and certainly the numbers of those units that can no longer be used because of a reduced quality of the RBCs. This is of course an interesting point in the whole discussion of blood conservation, mostly evaluated only on the percentage of patients without RBC transfusion and /or the number of units transfused.

The authors show that in a selected group of patients it is safe to perform cardiac surgery with the immediate availability of RBCs in the operating theatre. [5] Eighty-one percent of the patients did not receive any RBCs and the predictors for the need of blood transfusion are confirming the results of previous studies. [3, 4] However, some interesting points are not discussed in this study.

- Since the start of the NERC program, what was the percentage of RBCs returned to the blood bank and what was the percentage of units that could no longer be used because of reduced quality?
- It is emphasised that in all patients who need a blood transfusion, blood was available within 20 min after ordering it. However, was this really so? What was the time between ordering and delivering the blood in the operating theatre?
- What happens in cases of catastrophic bleeding problems, always possible even in the most simple cardiac procedure?

These questions are essential before general conclusions can be made about safety and the effectiveness of this new strategy, no immediate availability of red blood cells, in the NERC program.
